# Effects of Match Location, Match Status and Quality of Opposition on Regaining Possession in UEFA Champions League

**DOI:** 10.2478/hukin-2014-0048

**Published:** 2014-07-08

**Authors:** Carlos Humberto Almeida, António Paulo Ferreira, Anna Volossovitch

**Affiliations:** 1 SpertLab, Faculty of Human Kinetics, University of Lisbon, Estrada da Costa, 1495-688 Cruz Quebrada, Lisbon, Portugal.

**Keywords:** Soccer, notational analysis, situational variables, team performance, defensive strategies

## Abstract

The present study aimed to examine the independent and interactive effects of match location, match status, and quality of opposition on regaining possession, analysed by the type and zone of ball recovery, in matches played in the 2011–2012 UEFA Champions League. Twenty-eight matches of the knockout phase were evaluated post-event using a computerized notational analysis system. Multinomial logistic regression analysis was applied to identify the effects of the previously mentioned situational variables on ball recovery type and zone. Match status and quality of opposition main effects were observed for both dependent variables, while main effects of match location were only evident for ball recovery zone. Additionally, the interactions Match location ^*^ Quality of opposition and Match status ^*^ Quality of opposition were significant for both type and zone of ball recovery. Better teams employed more proactive defensive strategies, since, even when winning, they tried to sustain their defensive success on actions that aimed to gain the ball from the opponents. Results emphasized the tendency for home and losing teams to defend in more advanced pitch zones. Better-ranked teams were also more effective than worse-ranked teams in applying defensive pressure in more advanced pitch positions. The findings of the study suggest that the defensive strategies used by better teams imply more intense and organized collective processes in order to recover the ball directly from the opposing team. Furthermore, defending away from own goal and near the opponent’s one seems to be associated with success in elite soccer.

## Introduction

The available research regarding the analysis of match performance in soccer has been mainly conducted to examine the offensive phase of the game (Mackenzie and Cushion, 2013). It is well known that elite teams tend to base their competitive success on strategies that emphasize the maintenance of ball possession (Bloomfield et al., 2005; Lago and Martín, 2007; Lago, 2009). Nevertheless, being in possession is as important as recovering the ball when the opposing team attacks. Regaining possession occurs whenever a defender acts on the ball (or zone of the ball) in order to recover it from the opponents, initiating then attacking behaviours. In this sense, coaches and researchers should be concerned on how top teams regain possession and where their players put extra effort in trying to recover the ball in a specific match context (Barreira et al., 2011; Barreira et al., 2014).

Particularly in soccer, match location (playing at home or away), match status (whether the team is winning, drawing or losing), and quality of opposition (e.g. strong or weak) have been deemed as the most important situational influences on team performance during competition (Lago, 2009; Taylor et al., 2008; Taylor et al., 2010). Concerning match location, previous research (Lago-Peñas and Dellal, 2010; Lago-Peñas and Lago-Ballesteros, 2011; Gómez et al., 2012) has provided evidence of multiple home advantage effects on technical, tactical and strategic behaviours in professional soccer. For instance, Lago and Martín (2007), Lago (2009), and Lago-Peñas and Dellal (2010) demonstrated that playing at home increased ball possession compared to playing away. Home teams also tended to play more in offensive zones, performing a higher number of attacking actions (goals scored, shots on goal, passes, crosses, etc.), while more defensive behaviours (interceptions, clearances, etc.) were evident in less advanced pitch positions when playing away (Tucker et al., 2005; Taylor et al., 2010). Thus, home advantage can play an important role in determining the outcome of a match (Lago-Peñas and Lago-Ballesteros, 2011).

However, according to Lago (2009), the most important variable for explaining match possession and zones of play is the evolving score-line (i.e. match status). Indeed, several studies (Lago and Martín, 2007; Lago, 2009; Lago-Peñas and Dellal, 2010) have found that losing-match status was associated with greater ball possession. Authors suggested that this phenomenon could be explained by changes in strategies and styles of play adopted by teams according to the evolving score. When winning, teams decreased their possession, which indicated their preference for counterattacking or direct play; when losing, teams tried to regain possession in attacking zones and increased their possession, suggesting that they preferred to “control” the match by dictating play (Lago-Peñas and Dellal, 2010; Taylor et al., 2010; Ruiz-Ruiz et al., 2013). Despite that, in case of rounds with two-legged matches, as the knockout phase of UEFA competitions, researchers should consider not the evolving score-line of a unique match, but the aggregate score due to the effects of the first leg result on strategies employed by teams in the second leg (Page and Page, 2007). The quality of opposition is another situational factor that can affect teams’ performance (Taylor et al., 2008). Evidence indicates that stronger teams: a) dominated ball possession against their opponents (Bloomfield et al., 2005; Lago, 2009), b) demonstrated more stable patterns of play, independently of the evolving score-line (Lago, 2009; Lago-Peñas and Dellal, 2010), and c) did not experience the same home advantage as inferior opponents (Lago-Peñas and Lago-Ballesteros, 2011).

Since these situational factors might affect teams’ performance in different phases of play, the scarce number of studies that have investigated the effects of these factors on defensive performances in top-level soccer is surprising (Lago-Peñas et al., 2010), particularly in the most prestigious club competition in Europe: the UEFA Champions League (Lago-Peñas et al., 2011). Moreover, the existing research literature provides controversial and inconclusive results related to regaining possession according to playing zones. While some authors (Reilly and Gilbourne, 2003; Carling et al., 2005; Gómez et al., 2012) have argued that the chances to succeed increase when possession is regained in defensive and midfield areas, others (Garganta et al., 1997; Tenga et al., 2010; Lago-Ballesteros et al., 2012) have found higher performance efficiency in possessions regained in offensive zones.

The identification of players’ actions and corresponding pitch location (as some indirect tactical information) that lead to success in different competitive contexts may contribute to better understanding of the key factors that influence performance in soccer (Gómez et al., 2012). Therefore, the aim of this study was to examine the independent and interactive effects of match location, match status, and quality of opposition on regaining possession, analysed according to the type and zone of ball recovery, in matches played in the UEFA Champions League.

## Material and Methods

### Match sample

This study intended to investigate the defensive performance of the 16 most successful teams that participated in the 2011–2012 UEFA Champions League. Twenty-eight matches of the knockout phase (round of 16, quarter-finals, and semi-finals) of this competition were sampled from a total of 125 matches played. For each team that qualified for the round of 16, a minimum of 2 and a maximum of 6 matches were observed. To ensure equal representation of home and away matches for teams involved in a specific round, the periods of extra-time and penalty shootout were excluded from the sample; a total of 5457 regained possessions were analysed. Approval for the study was granted by the Ethics Committee of the Faculty of Human Kinetics, University of Lisbon.

### Variables and procedures

Matches were recorded from live TV broadcasts and converted to AVI format. In each match, selected actions were notated for both teams using the Match Vision Studio Premium v.1.0 software (Castellano et al., 2008), with data collection being based on independent and dependent variables. The independent and dependent variables used in the study are presented in Table 1.

An observational system – Foot-Ball Recovery Actions’ Observation System (Foot-BRAOS) – was developed for the recording, quantification and analysis of teams’ defensive actions that led to regaining possession. This system was supported by existing notational analysis literature and included performance indicators previously used in soccer performance analysis research. As the set of independent variables, performance indicators were recorded using Match Vision Studio Premium software.

A regained possession was observed whenever a player effectively recovered the ball from the opposing team. In this regard, for a ball recovery to be considered, one of the following criteria had to be noted in the early phase of the offensive sequence: (i) three consecutive touches on the ball, (ii) an accurate pass, (iii) a shot, (iv) a save by the goalkeeper, controlling the ball with the hands and/or maintaining possession, or (v) a ball received by a player after an interception, a tackle or an incomplete save by the goalkeeper. Regaining possession was analysed in terms of the type of ball recovery and the zone of the pitch in which it occurred. Five ball recovery types were considered: *interception*, *tackle*, *goalkeeper save*, *set play*, and *turnover won*; additionally, it was possible to identify ball recovery locations by dividing the pitch into 4 transverse zones with the same size – *defensive*, *defensive midfield*, *offensive midfield*, and *offensive* – as previously employed in other studies (Pratas et al., 2012; Lago-Ballesteros et al., 2012). A panel of independent soccer experts (1 experienced notational analysis researcher and 2 *UEFA Pro* coaches) were asked to assess content validity of Foot-BRAOS. After some minor changes in the system, all experts agreed with the definitions and the categories of proposed variables. Data collection was also ensured by 2 trained observers. Finally, coded data was exported into SPSS Statistics, version 19.0 (SPSS^®^ Inc., U.S.A.) for analysis.

### Reliability testing

Reliability was assessed through intraand inter-observer testing procedures. Intra-observer reliability was conducted by both observers notating data from four matches randomly selected from the sample. The matches were re-analysed after a 6-week period, to prevent any learning effect, and these data were compared with data from the original coding session. Subsequently, inter-observer reliability was assessed using data from the first coding session. The percentages of exact agreements between observations were determined using the Bellack’s formula (Van der Mars, 1989). Results showed high values of intra- (from 92.80 to 93.28%) and inter-observer agreement (from 88.68 to 90.79%). Weighted kappa (κ) was also calculated to eliminate the agreement by chance. Values ranged from 0.91 (ball recovery type) to 0.915 (ball recovery zone) for intra-observer reliability, and from 0.851 (ball recovery type) to 0.889 (ball recovery zone) for inter-observer reliability. These results indicated a very good strength of agreement (O’Donoghue, 2010).

### Statistical analysis

Multinomial logistic regression analysis was applied to estimate the probability of occurrence of the type and zone of ball recovery based on the values of match location, match status, and quality of opposition. These analyses break the dependent variables down into a series of comparisons between 2 categories, including the reference category (Field, 2009). The final models, which better fit the data, involved the main effects of predictor variables and the interaction terms that were entered into the estimated models following a stepwise procedure (i.e. only if the interactions were significant). The interactions Match location ^*^ Quality of opposition and Match status ^*^ Quality of opposition were added to both models. Regarding the type and zone of ball recovery, the interception and the defensive zone were chosen as reference categories, respectively. The level of statistical significance was set at p≤0.05.

## Results

### Descriptive analysis

A total of 5457 regained possessions were analysed. Table 2 shows the descriptive statistics concerning the type and zone of ball recovery in relation to match location, match status, and quality of opposition.

Overall, 2011 (36.9%) interceptions, 849 (15.6%) tackles, 139 (2.5%) goalkeeper saves, 1345 (24.6%) set plays, and 1113 (20.4%) turnovers won were registered. A total of 2631 (48.2%) regained possessions occurred in the defensive zone, 1790 (32.8%) in the defensive midfield, 884 (16.2%) in the offensive midfield, and 152 (2.8%) in the offensive zone.

### Multinomial logistic regression analysis

Due to the large number of associations, only results reaching statistical significance (p≤0.05) are presented. Table 3 displays regression coefficients, Wald statistics, odds ratios, and 95% confidence intervals for odds ratios for each predictor of the type of ball recovery.

The *Quality of opposition* significantly predicted whether teams recovered the ball through tackle or interception (p<0.01). The odds of similar-ranked teams to recover the ball through tackle rather than interception were 80.9% higher than for worse-ranked teams. The comparison between the categories goalkeeper save and interception revealed a significant effect of the interaction Match location ^*^ Quality of opposition (p<0.05). The odds ratio indicated that better-ranked teams were 93.8% less likely to regain possession through goalkeeper save than worse-ranked teams, when playing at home. The *Match status* and the interaction Match status ^*^ Quality of opposition significantly predicted whether teams regained possession through set play or interception (p<0.01 and p=0.001, respectively). The probability of winning teams to recover the ball through set play decreased 59.7% relatively to losing teams. Analyzing the data, we could also note that, when the play-off score was equalised, the chances of better-ranked teams to regain possession through set play were 66.7% lower than for worse-ranked.

We found significant associations between *Quality of opposition* and the interaction Match status ^*^ Quality of opposition and whether teams recovered the ball through turnover won or interception. Such fact was verified for *Quality of opposition* by comparing better-ranked to worse-ranked teams (p<0.01) and similar-ranked to worse-ranked teams (p<0.001). Better and similar-ranked teams were more likely to regain possession through turnover won compared to worse-ranked teams (140.4% and 124.6%, respectively). Significant effects were also observed comparing (1) better-ranked to worse-ranked teams, when both were winning (p<0.05), (2) similar-ranked to worse-ranked teams in combination with a winning-match status (p<0.05), and (3) similar-ranked to worse-ranked teams, when both were drawing (p<0.05). When winning, the probabilities for better and similar-ranked teams to regain possession through turnover won were lower (66.2% and 51.6%, respectively) than for worse-ranked ones. Instead, when drawing, similar-ranked teams were 38.3% less likely to regain possession through turnover won than worse-ranked ones.

Table 4 shows the independent and interactive effects of situational variables on the zone of ball recovery.

Regarding balls recovered in defensive midfield and offensive midfield zones, relatively to the defensive zone, significant differences were found between teams playing at home and away (p<0.001 and p<0.001, respectively). The odds of home teams regaining possession of the ball in defensive midfield and offensive midfield zones increased 103.7% and 134.2%, respectively, compared to visiting teams. The *Match status* was found to be an interesting predictor of the ball recovery zone. Significant effects were observed comparing drawing and losing teams (defensive midfield: p<0.001; offensive midfield: p<0.001). The odds of drawing teams regaining possession in the defensive midfield zone decreased by 43.6% compared to losing teams, and decreased by 52.1% if we consider the balls recovered in the offensive midfield. The *Quality of opposition* also predicted if teams regained possession in defensive midfield and offensive midfield zones rather than the defensive one. Significant effects were registered comparing the defensive performance of (1) similar to worse-ranked teams in the defensive midfield (p<0.05), (2) better to worse-ranked teams in the offensive midfield (p=0.01), and (3) similar to worse-ranked teams in the offensive midfield (p<0.01). The chances of similar-ranked teams regaining possession in the defensive midfield zone were 48.2% higher than for worse-ranked teams. Besides, better and similar-ranked teams were 116.8% and 91%, respectively, more likely than worse-ranked teams to regain possession in the offensive midfield.

*Match location* interacted with *Quality of opposition* to predict the chances of regaining possession in defensive midfield and offensive zones rather than the defensive zone. The number of balls recovered in the defensive midfield was significantly different comparing (1) better-ranked to worse-ranked home teams (p<0.05) and (2) similar-ranked to worse-ranked home teams (p<0.05). So, when playing at home, the odds of better and similar-ranked teams regaining possession in the defensive midfield were lower than for worse-ranked teams (41.1% and 40%, respectively). However, if we consider the possessions regained in the offensive zone, the probabilities change completely. Playing at home, better-ranked teams were 957.3% more likely to regain possession in the offensive zone than worse-ranked teams.

Finally, the interaction Match status ^*^ Quality of opposition significantly predicted where teams regained the ball possession. In terms of the defensive midfield, significant effects were found comparing (1) better-ranked to worse-ranked teams in combination with a winning-match status (p<0.01), (2) better-ranked to worse-ranked teams in combination with a drawing-match status (p<0.001), and (3) similar-ranked to worse-ranked teams when drawing (p<0.01). When ahead in the play-off round, the odds of better-ranked teams regaining possession in the defensive midfield zone were 168.1% higher than for worse-ranked teams. In turn, facing a drawing-match status, the odds of regaining possession in the defensive midfield increased 253% and 68%, respectively, for better and similar-ranked teams, compared to worse-ranked ones. In the offensive midfield, a significant difference was noted between better-ranked and worse-ranked teams in combination with a drawing-match status (p<0.05). When the round was tied, better-ranked teams were 119.4% more likely than worse-ranked to regain possession in the offensive midfield zone.

## Discussion

The aim of this study was to examine the independent and interactive effects of match location, match status, and quality of opposition on regaining possession, analysed by the type and zone of ball recovery, in the knockout phase of the UEFA Champions League. As mentioned before, very few studies have investigated the effects of situational variables on teams’ defensive performances in soccer (Lago-Peñas et al., 2010), particularly in the UEFA Champions League (Lago-Peñas et al., 2011).

As the results obtained by Barreira et al. (2014) showed, the interception was the type of ball recovery most executed in the sampled matches. This fact contradicts the findings of Rowlinson and O’Donoghue (2009), who reported higher values of tackles rather than interceptions in the knockout phase of the UEFA Champions League. Further research should be centred on this difference, which can be due to methodological discrepancies between studies. Multinomial logistic regression allowed the identification of several relevant trends related to the main and interaction effects of situational variables on the ball recovery type. First, when losing, teams regained more balls through set play. Since there is a need to create more goal-scoring opportunities, it is plausible to acknowledge increased defensive pressure during losing periods of the match (Lago, 2009; Ruiz-Ruiz et al., 2013), which forces the opponents to commit mistakes by, for example, throwing the ball out of play. However, this contrasts with the results of Taylor et al. (2008), since they did not find changes in the incidence of “set plays” as a function of match location, match status, and quality of opposition.

The quality of opposition influenced the ball recovery type as well. Similar-ranked teams were more effective than worse-ranked dispossessing (through tackling) the opponent attacker with the ball. The analysis of turnovers won indicates that the effectiveness of defensive strategies adopted by teams with better and similar-ranking is also influenced by their ability to force the opponents to play with no intention to keep possession or to make mistakes during the attack. For instance, Lago-Peñas and Dellal (2010) reported that better teams maintained a higher percentage of ball possession. Given their normal superiority, we suppose that they are not dependent on one single type of ball recovery. Besides, the evolving score-line affected the defensive performance of teams with distinct ranking. When ahead in the play-off, worse teams were more likely to recover the ball due to opponents’ turnovers; this result could be possibly explained by a worse team strategy that requires waiting for opponents’ mistakes. On the other hand, evidence reveals that better teams employed more proactive defensive strategies, since, even when winning or drawing, they tried to sustain their defensive success on actions that aimed to gain the ball directly from the opponents (interceptions and tackles), instead of waiting for their turnovers (Pratas et al., 2012; Ruiz-Ruiz et al., 2013).

The interaction between match location and quality of opposition indicated that, when playing at home, better teams presented more effective defensive methods, since they recovered the ball considerably less times due to their goalkeepers’ intervention. Indeed, some investigations (Tucker et al., 2005; Lago-Peñas and Lago-Ballesteros, 2011) found higher incidence of tackling actions during home matches. According to Pollard (2008), there are many factors that can improve team’s home performances. One possible explanation for these results is that home environment (i.e. crowd support) is associated with an increased functional aggressive response, which, consequently, enhances the effectiveness of defensive actions such as interceptions and tackles (Lago-Peñas and Lago-Ballesteros, 2011).

Teams regained possession more often in the defensive half of the pitch (81%), which concurs with data from other studies (Gréhaigne et al., 2002; Carling et al., 2005; Tenga et al., 2010; Gómez et al., 2012; Barreira et al., 2014). Statistical analysis showed that the ball recovery zone was influenced by situational variables, either independently or interactively. In line with Lago and Martín (2007), Taylor et al. (2008), and Lago (2009), the current research emphasizes that team strategies are influenced by match location, match status, and quality of opposition and teams change their playing style accordingly.

These findings confirm the existence of the home advantage effect on regaining possession in UEFA elite clubs. The tendency was for home teams to defend in more advanced pitch zones, which is, for some researchers (e.g. Garganta et al., 1997; Tenga et al., 2010; Lago-Ballesteros et al., 2012), a predictive of success in international competitions. This trend continued when teams were losing the play-off, and it was essential to equalise the score. In these circumstances, teams tended to move further up and regain possession in areas of the pitch closer to the opposing goal. Otherwise, when teams were drawing, they were more careful in their defensive approach. In order to avoid a goal from the opponent team (and concede advantage), the ball was recovered more regularly in the own defensive zone. Similar results were obtained by Lago-Ballesteros et al. (2012) concerning the odds of reaching the score-box according to match status. They verified that teams often showed a more defensive strategy when winning than losing, and vice-versa.

Curiously, when playing at home, the incidence of balls recovered in the defensive midfield was superior in worse-ranked teams. However, home teams with better UEFA ranking gained supremacy in terms of balls recovered in the offensive zone, which highlights a greater ability to regain possession in areas closer to the opposing goal. In general, better teams were much more efficient than worse teams in applying defensive pressure in more advanced zones of the pitch. Besides, when drawing or losing, the tendency was to move up in the pitch, regain possession and play more frequently in the opponent’s defensive half (Bloomfield et al., 2005; Taylor et al., 2008; Lago-Peñas and Dellal, 2010; Barreira et al., 2011). Data suggest that the defensive pressure employed by better teams is more likely to intensify as the match status becomes less favourable.

Overall, these findings support the recent critical review of Mackenzie and Cushion (2013), which highlights “regaining possession in the own final third” as one of the few aspects of defending play proposed in existing literature to influence “success” in soccer. Furthermore, by comparing the aggregate data of several teams, rather than analysing a single team’s success and failure, it is possible to obtain general values that can be used as normative data to improve teams’ performance in a collective way (Lago-Peñas et al., 2011). Our results enhance the knowledge regarding the influence of situational variables on the defensive performance of high-level teams. Coaches can use this information, in a strategic and tactical way, to prepare their teams for a specific competitive situation. Therefore, coaches should be aware that (1) promoting the intention to gain directly the ball from the opponents, and (2) pressurizing the opposing team near its goal seem to be the most appropriate defensive strategy to achieve success in elite soccer competitions.

Despite the discussion allowed by the obtained results, the regression models exhibited low-sized effect values. Nonetheless, we agree with Gómez et al. (2012) that this can be a reflection of soccer complexity. Given the sample size, it is hard to find models that use the minimum amount of independent variables to explain the dependent variables.

## Conclusions

Our findings suggest that match location, match status, and quality of opposition have independent and interactive effects on the defensive performance of club teams competing in the UEFA Champions League. The defensive strategies used by better teams imply more intense and organized collective processes in order to recover the ball directly from the opposing team. Additionally, defending away from the own goal and near the opponent’s one seems to be a factor associated with success in elite soccer. In order to better understand teams’ behaviours that lead to regaining possession, future research should focus on the interpersonal coordination between players in different competitive contexts, also taking into account the attacking team’s activity.

## Figures and Tables

**Table 1:** Categories of situational (independent) variables and performance indicators (dependent variables) and its definition and/or collection procedures

	Variables	Categories	Definition and/or collection procedures
**Independent**	Match Location	Home Away	Recorded as “home” or “away” depending on whether the sampled team was playing at its own ground or that of its opponent.
	Match Status	Winning Drawing Losing	Represents the evolving score of a round of two-legged matches when selected actions were recorded. Episodes were defined as “winning”, “drawing” or “losing” in relation to the number of goals scored and conceded by a team at the time of data entry (ahead, level or behind), and respecting the specific rules of UEFA competitions (e.g. away goals rule).
	Quality of Opposition	Better-ranked Similar-ranked Worse-ranked	Determined by the differences between the latest 2011–2012 UEFA rankings of opposing teams in each particular match (e.g. quarter-finals: SL Benfica [4] - Chelsea FC [5]; ranking difference = −1). A k-means cluster analysis was performed to identify a cut-off value of ranking differences and classify the quality of opposition. The grouping is done by minimizing the sum of squares of distances between data and the corresponding cluster centroid, which represents the arithmetic mean for each dimension separately over all the ranking differences in the cluster (Gómez et al., 2011). The results identified 3 clusters as follows: “better-ranked” (ranking differences between 4 and 15 points; n = 13), “similar-ranked” (ranking differences between −4 and 3 points; n = 30), and “worse-ranked” (ranking differences between −14 and −5 points; n = 13) teams.

**Dependent**	Ball Recovery Type	Interception	When the defender prevents a ball passed by an opponent from reaching its intended receiver by contacting the ball and keeping his own team in possession of the ball (Taylor et al., 2008; Rowlinson and O’Donoghue, 2009; Barreira et al., 2011; Barreira et al., 2014).
		Tackle	When the defender dispossesses the opponent of the ball through a physical challenge or defensive pressure (Taylor et al., 2008; Rowlinson and O’Donoghue, 2009; Barreira et al., 2011; Barreira et al., 2014).
		Goalkeeper Save	When the goalkeeper prevents the opposing team from scoring a goal after any kind of shot, i.e. a kick, a header or any intended deflection of the ball toward a goal (Barreira et al., 2011; Barreira et al., 2014).
		Set Play	Static situations deriving from opponents’ misses or fouls (goal kicks, thrown-ins, off-sides, and free kicks), and opponents’ goals (Barreira et al., 2011; Barreira et al., 2014).
		Turnover Won	When the defender collects, somewhere in the pitch, a ball lost (clearances or missed passes) by the opposing team (Gómez et al., 2012).
	Ball Recovery Zone	Defensive Defensive Midfield Offensive Midfield Offensive	Determined by dividing the pitch into 4 transverse zoneswith the same size.

**Table 2:** Absolute (and relative frequencies: %) of “ball recovery type” and “ball recovery zone” according to match location, match status and quality of opposition

Ball Recovery	Match Location		Match Status			Quality of Opposition		
	Home	Away	Winning	Drawing	Losing	Better	Similar	Worse
**Type**								
Interception	1004 (18.4)	1007 (18.5)	618 (11.3)	861 (15.8)	532 (9.7)	506 (9.3)	1012 (18.5)	493 (9.0)
Tackle	427 (7.8)	422 (7.7)	280 (5.1)	334 (6.1)	235 (4.3)	192 (3.5)	484 (8.9)	173 (3.2)
Goalkeeper Save	72 (1.3)	67 (1.2)	46 (0.8)	60 (1.1)	33 (0.6)	24 (0.4)	76 (1.4)	39 (0.7)
Set Play	629 (11.5)	716 (13.1)	349 (6.4)	524 (9.6)	472 (8.6)	303 (5.6)	692 (12.7)	350 (6.4)
Turnover Won	592 (10.8)	521 (9.5)	308 (5.6)	445 (8.2)	360 (6.6)	259 (4.7)	616 (11.3)	238 (4.4)
**Zone**																
Defensive	1287 (23.6)	1344 (24.6)	800 (14.7)	1076 (19.7)	755 (13.8)	532 (9.7)	1426 (26.1)	673 (12.3)
Defensive Midfield	901 (16.5)	889 (16.3)	542 (9.9)	744 (13.6)	504 (9.2)	477 (8.7)	923 (16.9)	390 (7.1)
Offensive Midfield	458 (8.4)	426 (7.8)	218 (4.0)	346 (6.3)	320 (5.9)	235 (4.3)	450 (8.2)	199 (3.6)
Offensive	78 (1.4)	74 (1.4)	41 (0.8)	58 (1.1)	53 (1.0)	40 (0.7)	81 (1.5)	31 (0.6)

**Table 3:** Multinomial logistic regression of “ball recovery type” as a function of situational variables

Ball Recovery Type	*B*	Wald	*P*	OR	95% CI
**Tackle vs. Interception**					
***Quality of Opposition***					
Similar-Ranked vs. Worse-Ranked	0.593	6.956	0.008	1.809	[1.164, 2.810]
**Goalkeeper Save vs. Interception**					
Match Location^*^Quality of Opposition					
Home^*^Better-Ranked vs. Home^*^Worse-Ranked	−2.77	5.704	0.017	0.062	[0.006, 0.608]
**Set Play vs. Interception**					
***Match Status***					
Winning vs. Losing	−0.909	6.931	0.008	0.403	[0.205, 0.793]
Match Status^*^Quality of Opposition					
Drawing^*^Better-Ranked vs. Drawing^*^Worse-Ranked	−1.10	11.672	0.001	0.333	[0.177, 0.626]
**Turnover Won vs. Interception**					
***Quality of Opposition***					
Better-Ranked vs. Worse-Ranked	0.877	6.964	0.008	2.404	[1.253, 4.611]
Similar-Ranked vs. Worse-Ranked	0.809	15.282	<0.001	2.246	[1.497, 3.370]
Match Status^*^Quality of Opposition					
Winning^*^Better-Ranked vs. Winning^*^Worse-Ranked	−1.09	6.168	0.013	0.338	[0.144, 0.796]
Winning^*^Similar-Ranked vs. Winning^*^Worse-Ranked	−0.726	5.287	0.021	0.484	[0.260, 0.898]
Drawing^*^Similar-Ranked vs. Drawing^*^Worse-Ranked	−0.483	4.325	0.038	0.617	[0.391, 0.973]

OR: Odds Ratio, CI: Confidence Interval.

Note: R^2^ = 0.025 (Cox & Snell), 0.027 (Nagelkerke). Model χ^2^(44) = 140.191.

**Table 4:** Multinomial logistic regression of “ball recovery zone” as a function of situational variables

Ball Recovery Zone	*B*	Wald	*P*	OR	95% CI
**Defensive Midfield vs. Defensive**					
***Match Location***					
Home vs. Away	0.712	14.535	<0.001	2.037	[1.413, 2.938]
***Match Status***					
Drawing vs. Losing	−0.573	12.824	<0.001	0.564	[0.412, 0.772]
***Quality of Opposition***					
Similar-Ranked vs. Worse-Ranked	0.393	6.036	0.014	1.482	[1.083, 2.027]
Match Location^*^Quality of Opposition					
Home^*^Better-Ranked vs. Home^*^Worse-Ranked	−0.530	4.118	0.042	0.589	[0.353, 0.982]
Home^*^Similar-Ranked vs. Home^*^Worse-Ranked	−0.510	6.158	0.013	0.600	[0.401, 0.898]
Match Status^*^Quality of Opposition					
Winning^*^Better-Ranked vs. Winning^*^Worse-Ranked	0.986	6.883	0.009	2.681	[1.283, 5.602]
Drawing^*^Better-Ranked vs. Drawing^*^Worse-Ranked	1.261	17.030	<0.001	3.530	[1.939, 6.426]
Drawing^*^Similar-Ranked vs. Drawing^*^Worse-Ranked	0.519	7.397	0.007	1.680	[1.156, 2.441]
**Offensive Midfield vs. Defensive**					
***Match Location***					
Home vs. Away	0.851	13.143	<0.001	2.342	[1.478, 3.709]
***Match Status***					
Drawing vs. Losing	−0.736	13.876	<0.001	0.479	[0.325, 0.706]
***Quality of Opposition***					
Better-Ranked vs. Worse-Ranked	0.774	6.585	0.010	2.168	[1.201, 3.914]
Similar-Ranked vs. Worse-Ranked	0.647	10.012	0.002	1.910	[1.279, 2.852]
Match Status^*^Quality of Opposition					
Drawing^*^Better-Ranked vs. Drawing^*^Worse-Ranked	0.786	5.559	0.018	2.194	[1.142, 4.216]
**Offensive vs. Defensive**					
Match Location^*^Quality of Opposition					
Home^*^Better-Ranked vs. Home^*^Worse-Ranked	2.358	6.038	0.014	10.573	[1.612, 69.36]

OR: Odds Ratio, CI: Confidence Interval.

Note: R^2^ = 0.028 (Cox & Snell), 0.031 (Nagelkerke). Model χ^2^(33) = 154.571.
